# Emotion regulation, psychological distress and demographic characteristics from an Ecuadorian sample: Data from the lockdown due to COVID-19

**DOI:** 10.1016/j.dib.2021.107182

**Published:** 2021-05-29

**Authors:** Jose A. Rodas, Maria Jara-Rizzo, Daniel Oleas

**Affiliations:** a University College Dublin, Ireland; b Universidad de Guayaquil, Ecuador; c Universidad Ecotec, Ecuador

**Keywords:** Psychological distress, Emotion regulation, COVID-19, Lockdown, Worries, Anxiety, Depression

## Abstract

Due to the rapid spread of COVID-19, several governments around the world implemented strict lockdown measures. However, these measures produced a number of negative psychological effects, such as increased anxiety and depression [1,2]. This article presents raw data from variables related to psychological distress, and from possible sources of psychological distress, such as the use of certain emotion regulation strategies, exposure to different media sources, demographic information (e.g., age, marital status, having children), or characteristics of the house (e.g., overcrowding or isolation). The data were collected online from March to June 2020 on an Ecuadorian sample of 618 participants (18–75 years old). The provided dataset could be useful to other researchers interested in investigating potential sources of psychological distress or vulnerable groups during a lockdown situation.

## Specifications Table

**Table:** 

Subject	Psychology
Specific subject area	Mental health, Clinical and Health Psychology
Type of data	Table
How data were acquired	Data were acquired using an online survey posted on Google Forms and advertised on social media.
Data format	Raw
Parameters for data collection	All data were collected online and anonymously. The study was advertised through social media (Facebook and Twitter). Participants were required to be Ecuadorian and at least 18 years old. After completing the online questionnaire, participants were encouraged to share the link of the study with relatives and friends.
Description of data collection	Data collection started on March 26 2020 and was closed on June 1 2020. It was advertised in Ecuador through social media (i.e. Facebook and Twitter) as a study investigating the effects of the lockdown measures on psychological distress. Participants were encouraged to share the link to the study.
Data source location	Institution: University of Guayaquil City: Guayaquil Country: Ecuador
Data accessibility	Repository name: Open Science Framework (OSF) Direct URL to data: https://osf.io/9cr3q/

## Value of the Data

• The data were collected during the first months of lockdown and includes an evaluation of psychological distress, cognitive emotion regulation strategies, hobbies, social support, seeking information related to COVID-19, perceived risk of infection, house characteristics, and demographic characteristics.
• Data can be used for identifying predictors of psychological distress during a lockdown situation.
• Other researches may look for group differences, identification of vulnerable groups, and the elaboration of models predicting psychological distress.
• Data can also be used to describe the lockdown situation in a developing South American country (Ecuador).

## 1 Data Description

The dataset presents the results from a survey including four questionnaires: (a) a General Questionnaire (the Supplementary Materials presents an English version of this questionnaire); (b) the State-Trait Anxiety Inventory (STAI); (c) the Cognitive Emotion Regulation Questionnaire (CERQ); and (d) the Center for Epidemiological Studies Depression scale (CES-D). All data is raw and is presented in a single dataset available at https://osf.io/9cr3q/. The STAI, CERQ, and CES-D required a total score to be calculated from their items. Total scores, as well as the score of each item, are provided. In cases where the score of an item required to be reversed, that is, converted into a different score in order to be used for the calculation of the total score, only the reversed score is presented.

Categorical variables including multiple options from the General Questionnaire were coded according to Table 1. In the Ecuadorian educational system a technical degree requires 3 years of formal study in an academic institution, a Bachelor's degree usually takes 4 years to complete and postgraduate degrees 12 to 18 months for masters and 4 years for doctoral studies. The last two options from marital status, civil union and living with a significant other, involves living with their couple, however a civil union in Ecuador involves a legal recognition of the couple with all the rights involved in marriage. Ecuador uses the US dollar as national currency and the basic salary by the time of assessment was of $400.

**Table 1:** Codes used in the dataset for categorical variables included in the General Questionnaire.

Variable	Code
**Level of education**	
Elementary	0
High school	1
Technical	2
Bachelor's degree	3
Postgraduate	4
**Marital status**	
Single	0
Married	1
Divorced	2
Civil union	3
Living with a significant other	4
**Employment situation**	
Unemployed	0
Informal work	1
Employee	2
**Family income**	
$0 - $400	0
$401 - $800	1
$801 - $1500	2
$1501 onward	3
**Where are you staying during the lockdown**	
Own house	1
House of a relative	2
House of a friend	3
House of a neighbor	4
Other	0
**Who takes care of the children**	
I do not have	0
Me	1
My partner	2
My partner and I	3
Other	4

Due to an error in data collection, data from the first 40 participants was lost for the CES-D.

The following set of tables describe the variables included in the dataset: Table 2 presents demographic information from the Ecuadorian sample in percentages and frequencies, Tables 3 and 4 presents descriptive statistics (means and standard deviations) from other variables collected in the General Questionnaire, and from the STAI, CERQ, and CES-D, respectively. Table 5 includes the medical and psychiatric diagnosis reported by participants, and Table 6 the sources of information commonly used by participants to obtain information about COVID-19. Both, Tables 5 and 6, present data in term of frequency.

**Table 2:** Demographic Characteristics of the 618 Ecuadorian participants.

Variable	%	Frequency
**Level of education**		
Primary school	0.32	2
High school	33.17	205
Technical degree	6.31	27
Undergraduate degree	44.34	274
Graduate degree	17.80	110
**Marital status**		
Single	65.50	402
Married	23.46	145
Divorced	6.31	39
Civil union	1.62	10
Living with a significant other	3.56	22
**Gender**		
Female	62.62	387
Male	36.25	224
Prefer not to say	1.13	7
**Have school-aged children**		
Yes	28.48	176
No	71.52	442
**Number of school-aged children**		
0	71.52	442
1	14.56	90
2	9.06	56
3	3.07	19
4	0.32	2
5	1.46	9
**Employment status**		
Unemployed	43.69	270
Casual	9.55	59
Employee	46.76	289
**Work in contact with others**		
Yes	17.96	111
No	82.04	507
**Family income per month (USD)**		
0-400	15.86	98
401-800	28.96	179
801-1500	30.74	190
1501 – or more	24.43	151
**Has been diagnosed of COVID-19**		
Yes	1.62	10
No	98.38	608

**Table 3:** Descriptive statistics from variables collected in the General Questionnaire.

Area	Mean	SD
Current worries*		
Home	3.83	1.31
Job	3.57	1.40
Money	3.85	1.14
Education	3.68	1.34
Health	4.40	0.91
Lack of social interaction	2.55	1.24
Income or health of friends or family	3.99	1.13
Areas thought to be affected in the future*		
Home	2.59	1.39
Job	3.58	1.36
Money	3.96	1.13
Education	3.43	1.36
Health	3.51	1.30
Lack of social interaction	2.60	1.32
Income or health of friends or family	3.75	1.21
**Other variables from the General Questionnaire**		
Number of persons in the house	4.23	1.96
Number of bedrooms in the house	3.35	1.41
Information seeking	2.86	4.06
Perceived risk of getting infected*	2.79	1.15
Number of hobbies	5.65	1.93
Perception on how appropriate is the lockdown measure*	4.41	0.92
Trust in information provided by the government*	2.30	1.08

⁎ Based on a Likert scale from 1 to 5

**Table 4:** Descriptive statistics from emotion regulation strategies, anxiety and depression questionnaires.

	Mean	SD
**CERQ**		
Self-blame	6.08	2.72
Acceptance	9.59	3.03
Rumination	8.11	3.10
Positive Refocusing	9.06	3.19
Refocus on Planning	10.21	3.12
Putting into Perspective	10.24	3.16
Catastrophising	6.55	2.88
Other-blame	5.87	2.78
Positive Reappraisal	10.62	3.18
**CES-D**		
Depression	19.81	11.48
**STAI**		
State Anxiety	26.54	11.83
Trait Anxiety	22.98	10.51

*Note.* CERQ = Cognitive Emotion Regulation Questionnaire; CES-D = Center for Epidemiological Studies Depression Scale; STAI = State-Trait Anxiety Inventory

**Table 5:** Medical and psychiatric diagnoses reported by the sample.

Diagnosis	Frequency
**Medical conditions**	
Allergies	16
Heart conditions	38
Diabetes	21
High cholesterol	57
Gastrointestinal diseases	108
Autoimmune diseases	42
Respiratory diseases	77
Other	37
None	323
**Psychiatric disorders**	
Depressive Disorders	26
Anxiety Disorders	34
Obsessive-Compulsive and Related Disorders	2
Other	6
None	556

*Note.* Participants could report more than one condition or disorder

**Table 6:** Sources of information consulted by participants for COVID-10 news.

Source of information	Frequency
TV news	479
Printed and digital press	171
Facebook	349
Twitter	188
Instagram	194
WhatsApp	152
Relatives	90
Radio	95
Youtube	48
Government	118
Other	31

*Note.* Participants could choose more than one.

**Fig. 1:**
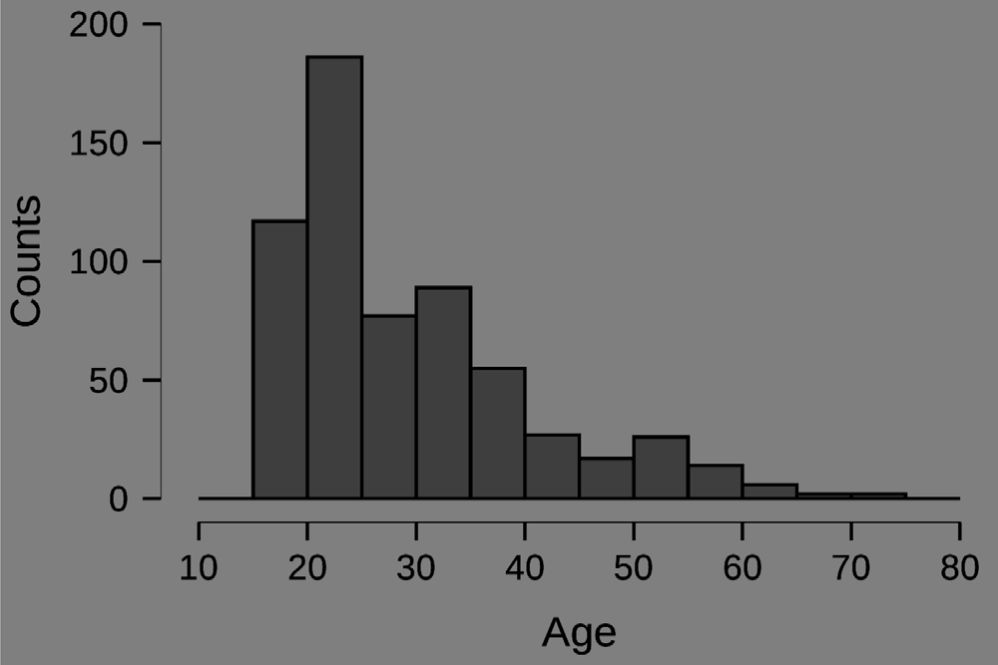
Age distribution of the 618 Ecuadorian participants.

Fig. 1 presents the age distribution of participants.

Fig. 1 presents a distribution plot of the participants’ age.

## 2 Experimental Design, Materials and Methods

The dataset contains data from four questionnaires and, although is not a representative sample, it includes participants from 60 different towns and cities from Ecuador.

The first questionnaire (General Questionnaire) covered demographic information, hobbies during the lockdown, medical and psychiatric history, characteristics of the house where the participant was staying during the lockdown, media sources usually accessed to look for information about COVID-19 (e.g. newspapers, TV news, WhatsApp, etc.), number of times they looked for information about COVID-19, and several variables evaluated using a 5-point Likert scale: trust in information provided by their government, perception about how appropriate was the lockdown measure, current worries, and areas to be affected after lockdown. This instrument took approximately 15 to 20 minutes to complete and can be found in the Supplemental Materials.

Emotion regulation was evaluated using the 27-item Spanish version of the Cognitive Emotion Regulation Questionnaire [3,4]. This instrument was designed to evaluate nine different emotion regulation strategies primarily relying on a cognitive component. The scale consists of 27 items presenting attitudes commonly used to cope with negative events. Participants were required to rate on a 5-point scale (1 = almost never, and 5 = almost always) each of the items and total scores were obtained for each sub-scale. Higher scores represent higher use of a particular strategy.

Anxiety was evaluated using the Spanish version of the State-Trait Anxiety Inventory [5]. This inventory consists of two sub-scales: State Anxiety, measuring a transitory experience of anxiety, and Trait Anxiety, measuring anxiety as a more stable characteristic in the person. Each scale includes 20 items describing different symptoms of anxiety and participants were required to rate how much they would experience these symptoms on a 4-point scale (0 to 3). A total score for each sub-scale was then calculated. Higher scores represent higher levels of anxiety.

Depression was evaluated using the Spanish version of the Center for Epidemiological Studies Depression Scale [6,7]. This is a 20-item scale covering different depression symptoms. Participants were required to rate on a 4-point scale (0 to 3) how much they experienced each of those symptoms during the previous week. A total score was obtained from all items. Higher scores represent higher levels of depression.

### 2.1 Procedure

Data collection was advertised through social media (i.e., Facebook and Twitter). Participants were provided with a link of a survey including all questionnaires and were encouraged to share it. The questionnaires were presented in the following order: (1) the General Questionnaire, (2) the State-Trait Anxiety Inventory, (3) the Cognitive Emotion Regulation Questionnaire, and (4) the Center of Epidemiological Studies Depression scale. In total, 618 Ecuadorian participants (mean age = 29.85, SD = 11.19 range = [18-75]) completed the survey from March 26 to June 1 from 2020.

## Ethics Statement

Informed consent was obtained from all participants. No identifiable information was collected. Ethical approval for data collection was granted by the Ethic's Committee from the Association of Clinical Psychologists of Tungurahua in Ecuador.

## CRediT Author Statement

**Jose A. Rodas:** Conceptualisation, Methodology, Data curation, Writing - review & editing; **Maria Jara-Rizzo:** Conceptualisation, Methodology, Data curation; **Daniel Oleas:** Conceptualisation, Data curation.

## Declaration of Competing Interest

The authors declare that they have no known competing financial interests or personal relationships which have or could be perceived to have influenced the work reported in this article.

## Appendix Supplementary materials

Image, application 1
