# Supplementary material for: Emotion regulation, psychological distress and demographic characteristics from an Ecuadorian sample: Data from the lockdown due to COVID-19
Source: Data Brief. 2021 May 29;37:107182. doi: 10.1016/j.dib.2021.107182 (PMC8181774; doi:10.1016/j.dib.2021.107182)
Supplement: Supplementary file 1 [file mmc1.docx]

**Supplemental Materials**

**General Questionnaire**

Questions with an asterisk (*) required an answer in order to continue.

**Start of the Questionnaire**

1. Do you agree to participate in the study*

Choose only one.

Yes, I agree

No, I preffer not to participate

**Dmographic information**

2. Country of residence _______________________

3. City of residence _______________________

4. Level of education *

Choose only one.

Elementary

High School

Technical

Bachelor’s degree

Postgraduate

5. Age _______________________

6. Marital status *

Choose only one.

Single

Married

Divorced

Civil union

Living with a significant other

7. Gender

Choose only one.

Woman

Man

I prefer not to say

8. Number of school-aged children *

Choose only one.

0

1

2

3

4

5 onward

9. Employment situation*

Choose only one.

Employee

Informal work

Unemployed

10. Are you currently working in contact with people? *

Choose only one.

Yes

No

11. Approximately. What is your family's income (adding the income of everyone who contributes to the house)?*

Choose only one.

$0-$400

$401-$800

$801-$1500

$1501 onwards

12. Do you have enough savings to survive these weeks? *

Choose only one.

Yes

No

From now on some questions will require you to answer using a scale from 1 to 5. Consider 1 as the minimum and 5 as the maximum. In these questions please choose the value that best represents your situation.

13. Have you been diagnosed with coronavirus disease? *

Choose only one.

Yes

No

14. If not, how much risk do you think you have of getting infected? *

Choose only one.

None 1 2 3 4 5 A lot risk

15. Before the health emergency due to COVID-19, were you diagnosed with any illness or psychological problem?

Choose only one.

Yes

No

16. If you answer yes to the previous question, indicate what the diagnosis was:

_______________________

17. Some close relative (grandparents, parents, siblings, first degree uncles or first degree cousins) have been diagnosed with a psychological illness.

Choose only one.

Yes

No

18. If you have any of the following medical conditions please select all that you have

Check all that apply.

Respiratory diseases

Gastrointestinal diseases

Diabetes

Hypertension

High cholesterol

Autoimmune diseases

Other:_______________________

Please answer the following questions on a scale of 1 to 5 (1 minimum and 5 maximum)

19. How appropriate do you think the “stay at home” measure taken by the Government is? *

Choose only one.

Not appropriate 1 2 3 4 5 Very appropriate

20. How much do you trust official government sources?? *

Choose only one.

Nothing 1 2 3 4 5 Completely

21. The house you are in during quarantine is: *

Choose only one.

Own

From relatives

From Friends

From Neighbors

Other:_______________________

22. How many people are living with you in the same house right now (counting you)? *

_______________________

23. ¿ How many rooms (with beds) does the house has? *

_______________________

24. How many bathrooms does the house has? *

_______________________

25. Which environments does the house has:

Check all that apply.

Backyard

Balcony

Living room

Dinning room

Kitchen room

Laundry room

Study / Office

26. Which of the following activities are you doing during lockdown? *

Check all that apply.

Reading

Play video game

Play tabletop games

Watch TV

Use social networks

Crafts

Use Streaming services (Youtube, Netflix, Hulu, etc)

Listen to music

Learn something

Play a musical instrument

Gambling

None

Other:_______________________

27. Who takes care of the children most of the time (in case you have)?

Choose only one.

I do not have

Me

My partner

My partner and I

Other:_______________________

28. Which of the following sources of information do you use to get the news about COVID-19? *

Check all that apply.

TV news

Written press

Facebook

Twitter

Instagram

Whatsapp

Ask Family

None

Other: _______________________

29. On a scale of 1 to 5, how much do you trust the information you receive from friends and acquaintances through social networks (WhatsApp, FaceBook, etc.)? *

Choose only one.

Nothing 1 2 3 4 5 Completely

30. How many times a day do you search for information about the current situation? *

_______________________

If you are using a mobile phone, please rotate it horizontally to have more space on the screen, otherwise you will not be able to see all the answer options.

On a scale from 1 to 5 (where 1 is nothing or almost nothing and 5 is much more than usual or the most) consider the following questions

31. Which of the following areas are you currently concerned about? *

Choose one option per row.

|  | 1 2 3 4 5 |
| --- | --- |
| Home |  |
| Job |  |
| Money |  |
| Education |  |
| Health |  |
| Lack of social interaction |  |
| Income or health of friends or family |  |

32. Which of the following areas do you think will be affected for you and your family after the quarantine ends? *

Choose one option per row.

|  | 1 2 3 4 5 |
| --- | --- |
| Home |  |
| Job |  |
| Money |  |
| Education |  |
| Health |  |
| Lack of social interaction |  |
| Income or health of friends or family |  |

The survey has ended

Thank you very much for your time. If you have finished completing the entire questionnaire please click on the "Submit" button on this page. If there is someone else near you who would like to complete the questionnaire feel free to send them the link to the surevey or you can fill out another questionnaire right here. Once you have clicked "Submit" choose the option "Submit another answer" on the next page.
